# Measurement of Heart Rate Using the Withings ScanWatch Device During Free-living Activities: Validation Study

**DOI:** 10.2196/34280

**Published:** 2022-09-01

**Authors:** Oonagh M Giggins, Julie Doyle, Suzanne Smith, Daniel R Crabtree, Matthew Fraser

**Affiliations:** 1 NetwellCASALA Dundalk Institute of Technology Dundalk Ireland; 2 Division of Biomedical Sciences University of the Highlands and Islands Inverness United Kingdom

**Keywords:** heart rate, photoplethysmography, PPG, wearable electronic device, wrist-worn device, validation study, heart, activity, physical activity, free-living activity

## Abstract

**Background:**

Wrist-worn devices that incorporate photoplethysmography (PPG) sensing represent an exciting means of measuring heart rate (HR). A number of studies have evaluated the accuracy of HR measurements produced by these devices in controlled laboratory environments. However, it is also important to establish the accuracy of measurements produced by these devices outside the laboratory, in real-world, consumer use conditions.

**Objective:**

This study sought to examine the accuracy of HR measurements produced by the Withings ScanWatch during free-living activities.

**Methods:**

A sample of convenience of 7 participants volunteered (3 male and 4 female; mean age 64, SD 10 years; mean height 164, SD 4 cm; mean weight 77, SD 16 kg) to take part in this real-world validation study. Participants were instructed to wear the ScanWatch for a 12-hour period on their nondominant wrist as they went about their day-to-day activities. A Polar H10 heart rate sensor was used as the criterion measure of HR. Participants used a study diary to document activities undertaken during the 12-hour study period. These activities were classified according to the 11 following domains: desk work, eat or drink, exercise, gardening, household activities, self-care, shopping, sitting, sleep, travel, and walking. Validity was assessed using the Bland-Altman analysis, concordance correlation coefficient (CCC), and mean absolute percentage error (MAPE).

**Results:**

Across all activity domains, the ScanWatch measured HR with MAPE values <10%, except for the shopping activity domain (MAPE=10.8%). The activity domains that were more sedentary in nature (eg, desk work, eat or drink, and sitting) produced the most accurate HR measurements with a small mean bias and MAPE values <5%. Moderate to strong correlations (CCC=0.526-0.783) were observed between devices for all activity domains, except during the walking activity domain, which demonstrated a weak correlation (CCC=0.164) between devices.

**Conclusions:**

The results of this study show that the ScanWatch measures HR with a degree of accuracy that is acceptable for general consumer use; however, it would not be suitable in circumstances where more accurate measurements of HR are required, such as in health care or in clinical trials. Overall, the ScanWatch was less accurate at measuring HR during ambulatory activities (eg, walking, gardening, and household activities) compared to more sedentary activities (eg, desk work, eat or drink, and sitting). Further larger-scale studies examining this device in different populations and during different activities are required.

## Introduction

The consumer wearable device industry has rapidly grown in recent years, with a wide range of devices available for monitoring and tracking physical activity [1]. In addition to providing physical activity metrics, such as step counts, distance covered, and energy expenditure, many devices now also incorporate an optical sensor that estimates the wearer’s heart rate (HR). These optical sensors estimate HR by means of a technique called photoplethysmography (PPG). This noninvasive optical technique detects blood volume changes in the microvascular bed of tissue beneath the skin [2]. Traditionally, wearable devices for monitoring HR have used an electrode-based chest strap to detect the electrical signals from the heart (eg, Polar). These devices have been extensively validated [3,4], and although much more convenient than an electrocardiogram (ECG) Holter monitor, these chest straps are not always feasible, desirable, or comfortable, particularly for prolonged use [5]. Therefore, wrist-worn measurement of HR via PPG represents a more unobtrusive and comfortable means of monitoring HR.

The potential to inform improved health care delivery is offered by wrist-worn PPG devices with screening or diagnostic, therapeutic monitoring, and self-management applications [6-9]. As the popularity and availability of PPG-enabled devices increases, it becomes more important that devices produce accurate measurements of HR, particularly if used in health care settings, where inaccurate readings could have detrimental consequences. Many studies have already been conducted examining the accuracy of HR measurements derived from wrist-worn devices equipped with PPG [10-13]. However, given the pace of technological development, these studies have largely been conducted on devices that are no longer available or that have been replaced by newer-generation models. Continual testing and validation of devices are, therefore, necessary to keep abreast of the advancements being made in this field. It is recommended that these devices should be evaluated in settings appropriate for their intended use [14]. Testing of devices in a controlled laboratory environment is initially required to establish validity of measurements. However, in laboratory evaluations, the range of activity types and intensities are often limited. Testing devices outside of the laboratory in more naturalistic environments during activities of daily living, where movements are more variable and sporadic, supports an understanding of how such devices are likely to perform in real-world context.

We have conducted this validation study to assess the accuracy of HR measurements produced by the Withings ScanWatch under free-living conditions. The Withings ScanWatch has an embedded PPG sensor for measuring HR as well as oxygen saturation, a triaxial accelerometer for monitoring activity, and electrodes for electrogram recording. To our knowledge, no study to date has examined HR measurements obtained from the ScanWatch, in the laboratory or in real-world conditions, as intended for general consumer use. Testing of the Withings ScanWatch was undertaken as part of evaluations of devices for use in trials conducted in our laboratory.

## Methods

### Study Design

A cross-sectional validation study was conducted to examine the accuracy of HR measurements obtained from the Withings ScanWatch. Data were collected from volunteer participants during free-living activities.

### Ethics Approval

The study protocol was approved by the Health and Science Ethics Committee in Dundalk Institute of Technology, and all procedures were conducted in accordance with the Declaration of Helsinki 1974 and its later amendments.

### Data Collection

A convenience sample of adult volunteers participated in the study. Participants were members of the NetwellCASALA Living Lab Panel in Dundalk Institute of Technology and were invited to participate via email and a newsletter sent to all panel participants (n=18). Informed consent was obtained from members upon enrolment to the Living Lab Panel, to take part in device testing research activities. Participants were provided with a study information leaflet and notified that they were not obliged to take part in this investigation and were free to withdraw from it at any point.

All panel members were eligible to participate. The only exclusion criteria that applied were the following: any cardiometabolic conditions, pacemakers, or implanted electrical devices that could interfere with HR measurements, as well undertaking any activities or work that could interfere with the completion of the study diary. Participants were screened by telephone to ensure suitability to take part in this investigation.

This study took place between April and June 2021, during level 5 of COVID-19 restrictions in Ireland. This prevented researcher visits to participants’ homes for technology deployment and data collection. Therefore, demographic data and Fitzpatrick skin tone measurements [15] were obtained verbally from participants via telephone. The study materials and required devices were delivered to participants’ homes by a member of the research team.

Participants were instructed via a Zoom videoconference call (Zoom Video Communications, Inc) on how to position the devices on their body for the study. The Withings ScanWatch was worn on participants’ nondominant wrist. A Polar H10 HR sensor (Polar Electro) was used as the criterion measure of HR. This type of monitor has been shown to be highly accurate in measuring HR [16]. The Polar chest strap was dampened and placed following Polar’s guidelines. Participants were also provided with written and pictorial instructions in the form of a study manual (Multimedia Appendix 1).

The Polar H10 was paired with the Polar Beat app on an Android smartphone for data acquisition, while the ScanWatch was paired with the Withings Health Mate app. Recordings commenced at 9 AM and continued for a 12-hour period until 9 PM. Participants were not asked to interact in any way with the watch or the Polar device during the 12-hour study period. Participants used a paper-based study diary to document all activities undertaken as well as the start and end times associated with each. Activities undertaken during the 12-hour period were classified according to the 11 following domains: (1) desk work—any activity sitting at a desk (eg, typing, emailing, writing, surfing the internet, phone calls, and video conferencing); (2) eat or drink (ie, sitting down for meals or snacks and drinks); (3) exercise—a defined and structured bout of moderate to intense physical activity (ie, jogging, running, and cycling); (4) gardening (eg, general garden maintenance, raking, weeding, and mowing the lawn); (5) household activities (ie, laundry, ironing, general home maintenance, vacuum cleaning, washing floors, washing windows, food preparation, cooking, setting table, and washing or putting away dishes); (6) self-care (eg, wash, dress, and care for self); (7) shopping (ie, purchasing goods or consuming other services in supermarkets and shops); (8) sitting—any period of quiet sitting activity, including watching TV, reading, and listening to music; (9) sleep (ie, any sleep and naps in bed, chairs, or reclined position); (10) travel (ie, travel by car, bus, and train); (11) walking—any purposeful walking activity of more than 1 minute duration (eg, walking the dog, walking to work, and walking to shop). The transitions between activities (eg, from sitting to standing or from standing to sitting) were not removed from the data, as per previous work [17].

### Data Processing

Following data collection, a member of the research team collected the devices and the completed study diary from participants. The ScanWatch was synchronized with the Withing’s Health Mate app, and the Polar Beat data was synchronized with the Polar Flow web application. Data were downloaded from both the Polar Flow and Health Mate web applications in CSV file format for analysis. The Polar H10 collected second-by-second HR data, whereas the ScanWatch measured HR approximately every 10 minutes. When in workout mode, the ScanWatch has a higher sampling frequency; however, in order to simulate real-world use, the workout mode was not used in this study. The Polar H10 data were plotted linearly to visually examine data quality and check for errors. Data from both devices were time-aligned and split according to their corresponding activity classification. Data alignment was performed by visually inspecting the data files using Microsoft Excel (Version 16) and manually matching the corresponding data points according to their timestamp. The timestamps used were provided via data export from the internal clock of each device, which was paired and synchronized with the same Android smartphone. The Polar H10 data were averaged in 60-second epochs, and comparisons between the ScanWatch and Polar H10 data were performed for each matched timestamp.

### Statistical Analysis

Descriptive statistics of the mean and SD were used to summarize all data collected. ScanWatch accuracy was assessed by calculating the difference between the ScanWatch-measured and the Polar H10–measured HR in beats per minute (BPM) for each activity domain. Mean absolute percentage error (MAPE) values were calculated as ([HRpolar – HRScanWatch] / HRpolar × 100). To assess the degree of agreement between the two devices, Lin’s Concordance Correlation Coefficient (CCC) and the 95% CI were calculated [14].

The strength of agreement was interpreted as follows: weak (CCC<0.5), moderate (CCC=0.5-0.7), and strong (CCC>0.7) [17].The Bland-Altman method was also used to express agreement between the Polar H10–measured and ScanWatch-measured HR, with limits of agreement (LoA) calculated as 1.96 times the SD of the mean difference [18].

## Results

A total of 6 Living Lab Panel members volunteered to take part in this study, and an additional 3 participants were recruited from within the research team. Due to a data syncing issue, Polar H10 data were missing for 2 participants, and therefore, data from 7 participants (3 male and 4 female; mean age 64, SD 10 years; mean height 164, SD 4 cm; mean weight 77, SD 16 kg; Fitzpatrick skin tone: n=2 for type II, n=3 for type III, and n=1 for type V) were analyzed. A number of ScanWatch data points were missing for each participant over the 12-hour study period, and therefore, the expected number of 504 data points was not achieved. A total of 422 matched timestamped data points from all participants were available for analysis. Table 1 outlines the number of data points analyzed for each activity domain. There were less than 10 matched data points available for analysis in the domains of exercise, self-care, and sleep. Data for these activity domains were therefore only descriptively analyzed. Correlation coefficients, mean bias, 95% LoA, and MAPE values across 12 hours and for each activity domain are also indicated in Table 1.

**Figure 1:**
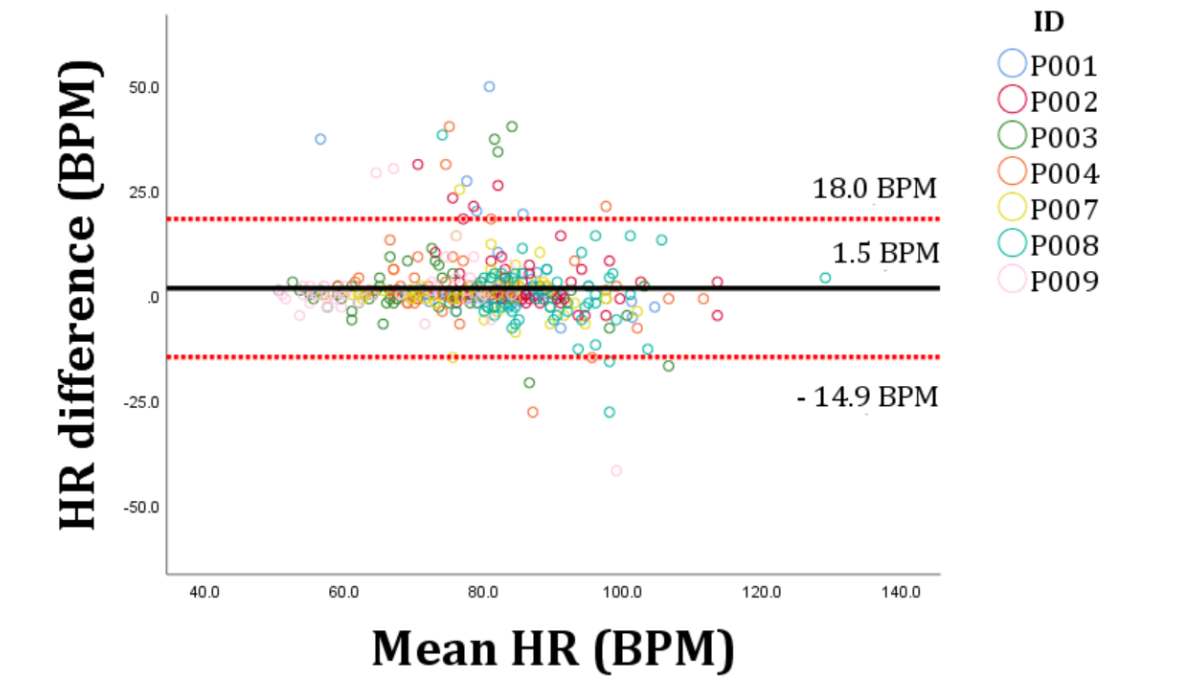
Bland-Altman plots outlining the agreement between the Withings ScanWatch and the Polar H10 over the 12-hour study period. The full horizontal line is the bias and the dotted lines are the 95% limits of agreement. Data are displayed in BPM. BPM: beats per minute; HR: heart rate.

Combined data across the 12 hours showed that the ScanWatch marginally underestimated HR with a mean bias of 1.5 (SD 8.4) BPM (95% LoA 18.0-14.9). There was a strong correlation between the ScanWatch-measured HR and the criterion device’s measured HR (CCC=0.796, 95% CI 0.759-0.828). The MAPE for the ScanWatch across the 12 hours was 5.1%. The distribution of the error is presented in the Bland-Altman plot in Figure 1.

**Figure 2:**
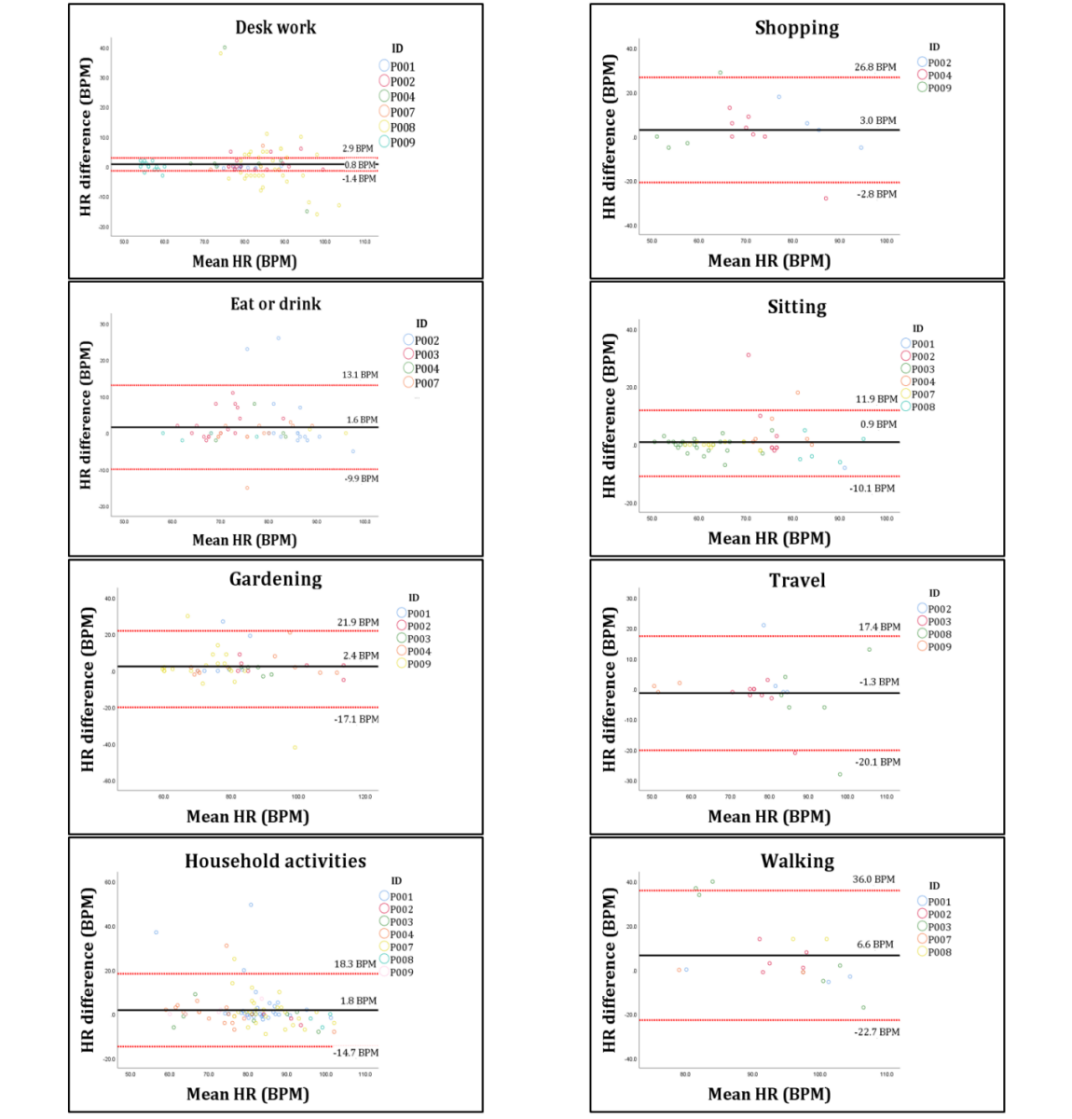
Bland-Altman plots outlining the agreement between the Withings ScanWatch and the Polar H10 for each activity domain. The full horizontal line is the bias and the dotted lines are the 95% limits of agreement. Data are displayed in BPM. BPM: beats per minute; HR: heart rate.

Figure 2 presents the Bland-Altman plots for all other activity domains. The activity domains that involved sitting, namely desk work, eat or drink, and sitting all produced a small mean bias and MAPE values <5% (desk work mean bias 0.8 (SD 1.1) BPM, MAPE=4.2%; eat or drink mean bias 1.6 (SD 5.9) BPM, MAPE=3.9%; and sitting mean bias 0.9 (SD 5.6) BPM, MAPE=3.8%). Strong correlations were observed for the desk work (CCC=0.827, 95% CI 0.748-0.883), eat or drink (CCC=0.796, 95% CI 0.678-0.875), and sitting (CCC=0.865, 95% CI 0.783-0.918) activity domains. All other activity domains produced MAPE values <10%, except for shopping (Table 1). The ScanWatch exhibited a weak correlation with the criterion measure of HR during the walking activity domain, underestimating HR with a mean bias of 6.6 (SD 15.0) BPM. During the activity domains gardening, household activities, and shopping, the ScanWatch had a tendency to underestimate HR, with fair to moderate correlations observed. The ScanWatch overestimated HR during the travel domain with a mean bias of –1.3 (SD 9.5) BPM and MAPE of 6.5%.

**Table 1:** Validity of measuring heart rate using the Withings ScanWatch.

Activity domain	Data points^a^, n	Polar H10, mean (SD)	Withings ScanWatch				
			Mean (SD)	Mean bias (SD)	95% LoA^b^ (upper, lower)	MAPE^c^ (%)	CCC^d^ (95% CI)
12-hours	422	79.2 (12.9)	77.7 (13.7)	1.5 (8.4)	18.0, –14.9	5.1	0.796 (0.759-0.828)
Desk work	87	79.5 (12.1)	78.8 (13.1)	0.8 (1.1)	2.9, –1.4	4.2	0.827 (0.748-0.883)
Eat or drink	56	77.7 (9.5)	76.1 (9.5)	1.6 (5.9)	13.1, –9.9	3.9	0.796 (0.678-0.875)
Exercise	3	96.3 (30.0)	94.3 (28.3)	—^e^	—	—	—
Gardening	48	82.3 (14.0)	79.9 (15.5)	2.4 (10.0)	21.9, –17.1	6.3	0.762 (0.617-0.858)
Household activities	100	81.7 (9.9)	79.9 (12.0)	1.8 (8.4)	18.3, –14.7	5	0.696 (0.586-0.782)
Self-care	5	88.6 (11.2)	91.6 (11.2)	—	—	—	—
Shopping	16	72.8 (12.2)	69.8 (14.7)	3.0 (12.1)	26.8, –20.8	10.8	0.582 (0.165-0.822)
Sitting	58	68.6 (11.0)	67.7 (11.0)	0.9 (5.6)	11.9, –10.1	3.8	0.865 (0.783-0.918)
Sleeping	7	69.5 (1.4)	69.4 (2.1)	—	—	—	—
Travel	22	78.1 (13.1)	79.5 (14.9)	–1.3 (9.5)	17.4, –20.1	6.5	0.764 (0.526-0.892)
Walking	20	97.4 (7.2)	90.8 (14.4)	6.6 (15.0)	36.0, –22.7	10	0.164 (–0.134-0.435)

^a^Number of data points analyzed for each domain.

^b^LoA: limits of agreement.

^c^MAPE: mean absolute percentage error.

^d^CCC: concordance correlation coefficient.

^e^Not applicable.

## Discussion

### Principal Findings

Wearable activity monitoring devices have attracted considerable interest over the past few years [1]. Many of these devices can now measure HR using PPG technology. Wrist-worn PPG-enabled devices have several advantages over traditional ECG-based methods of measuring HR and provide a valuable means of estimating physical activity intensity. This ecological validation study sought to assess the accuracy of HR measures produced by the Withings ScanWatch, in a sample of adult volunteers, under free-living conditions. Overall, across the 12-hour study period, the ScanWatch produced accurate measurements of HR, performing within an acceptable error range for measuring HR (MAPE <10%) [14]. This error range is acceptable for a device intended for general consumer use; however, it would not be acceptable in settings such as health care or clinical trials where accurate measurements are paramount.

### Comparison to Prior Work

Numerous studies have examined the accuracy of HR measures produced by wrist-worn PPG-enabled devices [19-24]. A recent systematic review and meta-analysis synthesized the evidence from these studies to determine the overall validity of HR measured by such devices [13]; in this review, 15 different device brands were evaluated, with devices from Fitbit, Apple, Garmin, Mio, and TomTom being the most frequently studied. Most trials included in this review were conducted in controlled laboratory environments with activity types such as treadmill exercise, elliptical exercise, resistance training, biking on cycle ergometer, and daily living activities being evaluated. Overall, the pooled estimates in this systematic review indicate that wrist-worn estimates of HR measurements closely resemble HR derived from the criterion measure of HR, with only two activity types—resistance training and cycling—showing sizeable differences between wrist-worn devices and the criterion measure of HR.

To the authors’ knowledge, only one previous study has been conducted to assess the accuracy of HR measurements produced by devices outside laboratory and in more naturalistic environments during activities of daily living, where movements are more variable [17]. This study [17] assessed the accuracy of HR measurements produced by the Apple Watch 3 and the Fitbit Charge 3, as compared with the gold standard reference method of a 3-lead ECG, under free-living conditions, and showed that these devices were generally accurate across the 24-hour study period, with MAPE values of 5.86% and 5.96%, respectively. In our investigation, the MAPE of the ScanWatch across the 12-hour recording period was 5.1%. The ScanWatch produced accurate measurements of HR during the lower intensity activity domains of sitting, desk work, and eat or drink, with MAPE values <5% compared to the Polar H10. The ScanWatch’s estimation of HR was less accurate during activities that may have involved more wrist movement. Motion artifact is commonly cited as a source of measurement error in wrist-worn PPG-enabled measurement of HR [25]. Motion artifacts have long posed a problem with the measurement of physiological signals [26], with the contamination of PPG signals usually caused by movement of the wrist or hand. In this study, participants were instructed to wear the ScanWatch on their nondominant wrist, which may have resulted in fewer erratic movements during these activities, and therefore, the impact of the motion artifact may have been limited. Future studies should examine the differences in HR measurements produced when the device is worn on dominant versus the nondominant wrist.

The results of our study revealed that the ScanWatch produced less accurate measurements of HR with increasing intensity of activity. The ScanWatch underestimated HR during the higher-intensity activity domains of gardening, household activities, walking, and shopping. The highest MAPE was observed during the walking and shopping activity domains (10% and 10.8%, respectively). These findings are similar to those of previously conducted research [27,28], which showed that increasing exercise intensity and increasing arm movement during household tasks can interfere with PPG measurement of HR. The ScanWatch overestimated HR measurements during the travel activity domain. Participants may have classified the walk to and from their mode of transport as part of the travel activity domain, thereby providing a possible explanation for the overestimation of HR measurements during the largely sedentary activity of travel. Not excluding transition periods between activities in this study, it was not possible to assess if this was the explanation for the readings. Furthermore, as with household tasks [20,21], arm movements while driving may have resulted in motion artefact that may have contaminated readings. Further study would be required to explore this possibility.

Although the overall measurements of HR produced by the ScanWatch in the study are within an acceptable error range [17], a degree of caution should be applied when selecting this device for the measurement of HR. As the ScanWatch appears to underestimate HR during higher-intensity activities, further evaluations are recommended to examine how accurate this device is during different types and intensities of exercise. Nonetheless, for everyday activities, the ScanWatch offers a practical solution for estimating HR.

### Strengths and Limitations

To our knowledge, this is the first study examining the ecological validity of HR measurements produced by the ScanWatch, when used during daily activities, as intended for consumers in real-world contexts. However, there are some limitations to this study. First, the main limitation of this study is the difference between the ScanWatch and the Polar H10 in terms of the sampling rate employed. The Polar H10 collected data at a frequency of 1 Hz, whereas the ScanWatch sampled HR data approximately every 10 minutes. To address this, the Polar H10 data were averaged, and comparisons were performed between matched timestamps. Nonetheless, as the ScanWatch only measures HR every 10 minutes, short-term changes in HR could not be captured. This renders the device inappropriate in situations where continuous measurements of HR are required, as in health care.

Second, the study included a small sample of adults; therefore, the results cannot be generalized to any particular cohort. Future studies should include larger number of participants. In addition, the impact that demographic variables such as skin tone have on the accuracy of measurements should also be evaluated. Previous studies have shown that darker skin tones absorb more green light, which can be problematic for PPG devices using green LED light [29]. Although recent work examining the role of skin tone on wrist-worn HR devices found no significant difference in accuracy across skin tones [25], future work should continue to explore the accuracy of wrist-worn device measurements across different skin tones.

Third, due to the COVID-19 restrictions in place during the time of data collection, we had little control or oversight of how participants positioned and wore the ScanWatch and the Polar H10 devices during the study period. Participants were given written and pictorial instructions in the form of a study manual and verbal instructions via a Zoom videoconference call to guide correct device positioning, but there may have been inconsistencies in device placement between participants. Nevertheless, this reflects real-world usage of devices where the consumer independently dons a device.

Fourth, in this study, participants’ self-report of activities undertaken during the 12-hour study period was used. There may have been discrepancies in how participants reported some activities and how they documented the start and end time of the activity. Previous research has demonstrated that diary recall within physical activity can result in inaccuracy and bias [30]. Future investigations could make use of body-worn cameras to record the activities undertaken by participants during free-living activities [31]. In addition, the transition times between activities were not excluded from the data, and this may have resulted in some data points being misclassified. The free-living nature of this study resulted in some activity domains with very few data points available, and this data could only be analyzed descriptively.

Finally, the Polar H10 was used to provide the criterion measure of HR in this study. A 12-lead ECG is considered the gold standard reference measurement of HR; however, it would not have been feasible to use it in this investigation. The Polar H10 does not allow for the export of raw ECG data. The signal could therefore not be checked for noise, and this must also be acknowledged as a limitation of the study.

### Conclusions

Wrist-worn devices that incorporate PPG sensing represent an exciting means of measuring HR. This study examined the accuracy of HR measurements produced by the Withings ScanWatch during free-living activities in a sample of adult volunteers. The results revealed that the ScanWatch measured HR with acceptable accuracy during a range of day-to-day activities in a small cohort of healthy, largely sedentary, middle-aged adults. The findings indicate that this device is suitable for general consumer use. However, further investigations examining HR measurements during activities with more vigorous intensity are required before definitive conclusions on device accuracy can be made.

## Appendix

Multimedia Appendix 1 Study manual.

Multimedia Appendix 2 Data availability.

## Abbreviations

BPM: beats per minute
CCC: concordance correlation coefficient
ECG: electrocardiogram
HR: heart rate
LoA: limits of agreement
MAPE: mean absolute percentage error
PPG: photoplethysmography

## Data Availability

The data that support the findings of this study are available in Multimedia Appendix 2.
