# Supplementary material for: Measurement of Heart Rate Using the Withings ScanWatch Device During Free-living Activities: Validation Study
Source: JMIR Form Res. 2022 Sep 1;6(9):e34280. doi: 10.2196/34280 (PMC9478823; doi:10.2196/34280)
Supplement: Multimedia Appendix 1 [file formative_v6i9e34280_app1.docx]

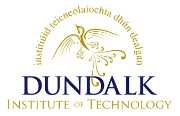

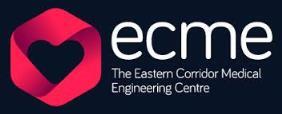

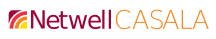


**Measurement of Heart Rate Using the Withings ScanWatch Device during Free-Living Activities: A Validation Study**

**Study Manual**

# Welcome

Thank you for agreeing to take part in our study. In this study we will examine the accuracy of heart rate measurements produced by a wristwatch, called the Withings ScanWatch.

You will need to wear this wristwatch and a chest strap to measure your heart rate for 12-hours. Both the wristwatch and the chest strap need to be worn continuously. They should only be removed for showering/bathing.

You will also need to take note of everything you do during the 12-hour study period. You will record this information using the study diary provided. This information is important as it will allow the research team to assess heart rate measurements during different activities.

A member of the research team will contact you and will schedule your study day at a time that is convenient to you.

On your scheduled study day, a member of the research team will video call you, to help you get started and explain everything you will need to do. This details of this video call will be sent to you via email.

During the 12-hour study day, you should continue with your daily routines and activities as normal.

Please read in advance the information in this study guide carefully and discuss any queries/concerns with a member of the research team.

# Study Pack

The following items are included in your study pack, which you will have received along with this guide;

- **Withings Scanwatch,** charging cable, quick installation guide and carry case.


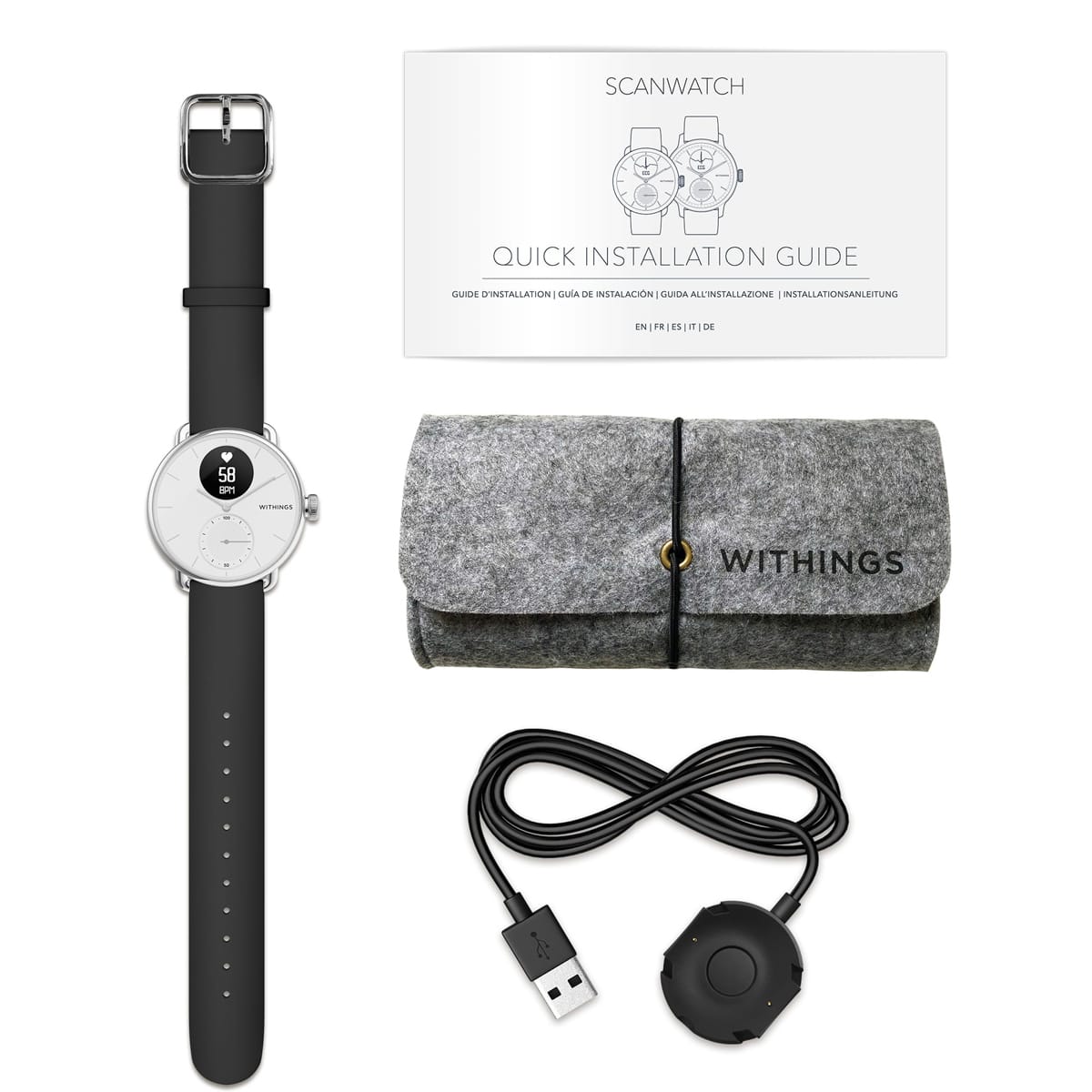


- Polar H10 Chest strap


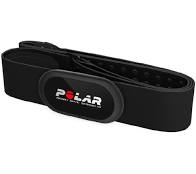


- A Samsung phone
- An Activity Diary

**Please check that you have received all the items above in your study pack. Notify the research team immediately if any item is missing.**

## On your scheduled study day, a member of the research team will video call you and will talk you through the information below.

### Withings ScanWatch

The wristwatch should be worn around your non-dominant wrist (i.e. the side least preferred for handwriting). The watch should be tightened around your wrist, a finger’s breath above your wrist bone (see figure below). The wristwatch should feel tight but comfortable.


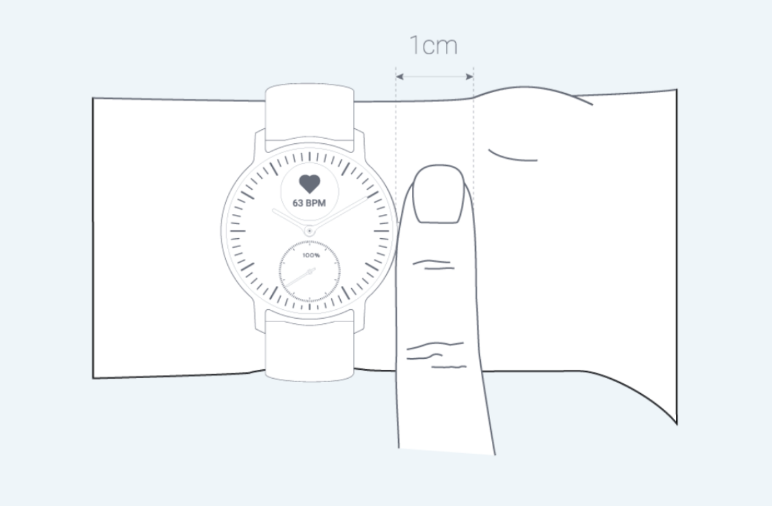


#### Charging

The watch should not require charging during the study period. However in the event that it does please refer to the Scanwatch ‘Quick Installation Guide’ enclosed in the pack for instructions on how to charge the device.

#### Cleaning

If needed, use a lint-free cloth moistened with warm water to clean the watch. All equipment was fully sanitised before being sent to you and will be fully sanitised upon return.

### Polar H10 Strap

The Polar H10 Strap is worn around your chest;

- The plastic electrode areas on the reverse side of the strap detect your heart rate.


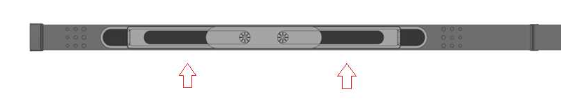


- The connector sends heart rate signals to the phone.


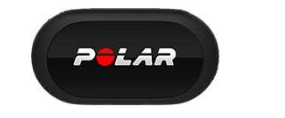


Before wearing, the plastic electrode areas need to be moistened slightly.


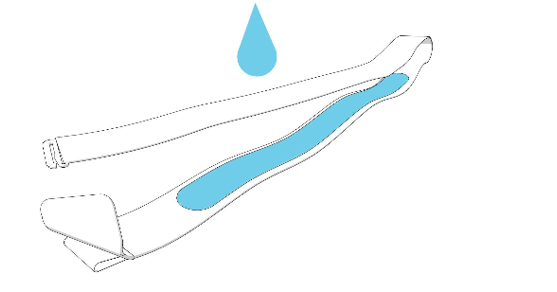


Fasten the strap around your chest and adjust to fit snugly. Attach the connector to the strap.


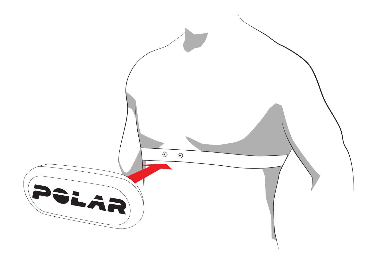


The polar H10 will be delivered fully charged and will not require charging during the study.

#### To start recording on Polar H10

Power on phone and open the Polar Beat on the home screen


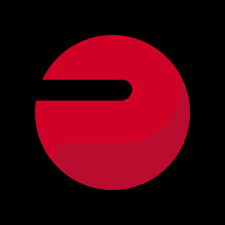


Press Start to begin recording.


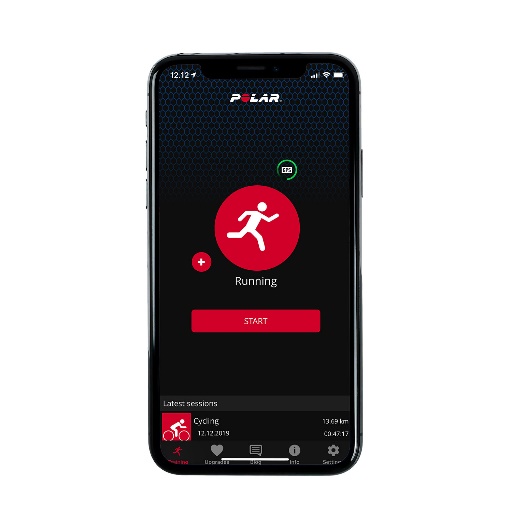


Once recording has started, press lock button on phone.


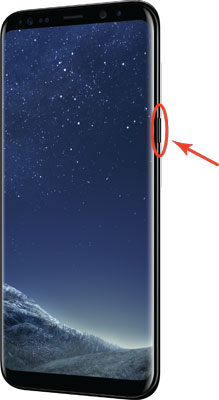


**The phone must be kept in close proximity to the Polar strap to pick up the heart rate signals** **throughout the 12-hour study period.** You can carry the phone in your pocket/bag during the day. At rest/sitting the phone can be removed from your pocket, but ensure it is always placed close by and always in the same room as you.

**Please ensure the study phone is fully charged prior to the study day.**

# Activity Diary

**Please use this diary to record all activities that you complete during the 24 hour study day.**

**Record the start and end time of each activity.**

**Activities can be recorded according to the following activity domains;**

| **Activity Domain** | **Description** |
| --- | --- |
| Desk work | Any activity sitting at a desk, e.g. typing, e-mailing, writing, surfing the internet, phone calls, video conferencing. |
| Eat/Drink | Sitting down for meals/snacks and drinks. |
| Exercise | A defined and structured bout of moderate-intense physical activity, e.g. jogging, running, cycling. |
| Gardening | General garden maintenance, raking, weeding, mowing the lawn. |
| Household | Laundry, ironing, general home maintenance, vacuum cleaning, washing floors, washing windows, food preparation, cooking, setting table, wash/put away dishes, etc. |
| Self-care | Wash, dress, care for self. |
| Shopping | Purchase goods/consume other services in supermarket/shop. |
| Sitting | Any period of quiet sitting activity, including watching TV, reading listening to music. |
| Sleep | Any sleep and naps in bed, chair or reclined position |
| Travel | Travel by car, bus, train. |
| Walking | Any purposeful walking activity of more than 1 minute duration, e.g. walking the dog, walking to work, walking to shop. |

| **Time** | | **Activity Domain** | **Notes** |
| --- | --- | --- | --- |
| **Start** | **End** |  |  |
|  |  |  |  |
|  |  |  |  |
|  |  |  |  |
|  |  |  |  |
|  |  |  |  |
|  |  |  |  |
|  |  |  |  |
|  |  |  |  |
|  |  |  |  |
|  |  |  |  |
|  |  |  |  |
|  |  |  |  |
|  |  |  |  |
|  |  |  |  |
|  |  |  |  |
|  |  |  |  |
|  |  |  |  |
|  |  |  |  |
|  |  |  |  |
|  |  |  |  |
|  |  |  |  |
|  |  |  |  |
|  |  |  |  |
|  |  |  |  |
|  |  |  |  |
|  |  |  |  |
|  |  |  |  |
|  |  |  |  |
|  |  |  |  |
|  |  |  |  |
|  |  |  |  |
|  |  |  |  |
|  |  |  |  |
|  |  |  |  |
|  |  |  |  |
|  |  |  |  |
|  |  |  |  |
|  |  |  |  |
|  |  |  |  |
|  |  |  |  |
|  |  |  |  |
|  |  |  |  |
|  |  |  |  |
|  |  |  |  |
|  |  |  |  |
|  |  |  |  |
|  |  |  |  |
|  |  |  |  |
|  |  |  |  |
|  |  |  |  |
|  |  |  |  |
|  |  |  |  |
|  |  |  |  |
|  |  |  |  |
|  |  |  |  |
|  |  |  |  |
|  |  |  |  |
|  |  |  |  |
|  |  |  |  |
|  |  |  |  |
|  |  |  |  |
|  |  |  |  |
|  |  |  |  |
|  |  |  |  |
|  |  |  |  |
|  |  |  |  |
|  |  |  |  |
|  |  |  |  |
|  |  |  |  |
|  |  |  |  |
|  |  |  |  |
|  |  |  |  |
|  |  |  |  |
|  |  |  |  |
|  |  |  |  |
|  |  |  |  |
|  |  |  |  |
|  |  |  |  |
|  |  |  |  |
|  |  |  |  |
|  |  |  |  |
|  |  |  |  |
|  |  |  |  |
|  |  |  |  |
|  |  |  |  |
|  |  |  |  |
|  |  |  |  |
|  |  |  |  |
|  |  |  |  |
|  |  |  |  |
|  |  |  |  |
|  |  |  |  |
|  |  |  |  |
|  |  |  |  |
|  |  |  |  |
|  |  |  |  |
|  |  |  |  |
|  |  |  |  |
|  |  |  |  |
|  |  |  |  |
|  |  |  |  |
|  |  |  |  |
|  |  |  |  |
|  |  |  |  |
|  |  |  |  |
|  |  |  |  |
|  |  |  |  |
|  |  |  |  |
|  |  |  |  |
|  |  |  |  |
|  |  |  |  |
|  |  |  |  |
|  |  |  |  |
|  |  |  |  |
|  |  |  |  |
|  |  |  |  |
|  |  |  |  |
|  |  |  |  |
|  |  |  |  |
|  |  |  |  |
|  |  |  |  |
|  |  |  |  |
|  |  |  |  |
|  |  |  |  |
|  |  |  |  |
|  |  |  |  |
|  |  |  |  |
|  |  |  |  |
|  |  |  |  |
|  |  |  |  |
|  |  |  |  |
|  |  |  |  |
|  |  |  |  |
|  |  |  |  |
|  |  |  |  |
|  |  |  |  |
|  |  |  |  |
|  |  |  |  |
|  |  |  |  |
|  |  |  |  |
|  |  |  |  |
|  |  |  |  |
|  |  |  |  |
|  |  |  |  |
|  |  |  |  |
|  |  |  |  |
|  |  |  |  |
|  |  |  |  |
|  |  |  |  |
|  |  |  |  |
|  |  |  |  |
|  |  |  |  |
|  |  |  |  |
|  |  |  |  |
|  |  |  |  |
|  |  |  |  |
|  |  |  |  |
|  |  |  |  |
|  |  |  |  |
|  |  |  |  |
|  |  |  |  |
|  |  |  |  |
|  |  |  |  |
|  |  |  |  |
|  |  |  |  |
|  |  |  |  |
|  |  |  |  |
|  |  |  |  |
|  |  |  |  |
|  |  |  |  |
|  |  |  |  |
|  |  |  |  |
|  |  |  |  |
|  |  |  |  |
|  |  |  |  |
|  |  |  |  |
|  |  |  |  |
|  |  |  |  |
|  |  |  |  |
|  |  |  |  |
|  |  |  |  |
|  |  |  |  |
|  |  |  |  |
|  |  |  |  |
|  |  |  |  |
|  |  |  |  |
|  |  |  |  |
|  |  |  |  |
|  |  |  |  |
|  |  |  |  |
|  |  |  |  |
|  |  |  |  |
|  |  |  |  |
|  |  |  |  |
|  |  |  |  |
|  |  |  |  |
|  |  |  |  |
|  |  |  |  |
|  |  |  |  |
|  |  |  |  |
|  |  |  |  |
|  |  |  |  |
|  |  |  |  |
|  |  |  |  |
|  |  |  |  |
|  |  |  |  |
|  |  |  |  |
|  |  |  |  |
|  |  |  |  |
|  |  |  |  |
|  |  |  |  |
|  |  |  |  |
|  |  |  |  |
|  |  |  |  |
|  |  |  |  |
|  |  |  |  |
|  |  |  |  |
|  |  |  |  |
|  |  |  |  |
|  |  |  |  |
|  |  |  |  |
|  |  |  |  |
|  |  |  |  |
|  |  |  |  |
|  |  |  |  |
|  |  |  |  |
|  |  |  |  |
|  |  |  |  |
|  |  |  |  |
|  |  |  |  |
|  |  |  |  |
|  |  |  |  |
|  |  |  |  |
|  |  |  |  |
|  |  |  |  |
|  |  |  |  |
|  |  |  |  |
|  |  |  |  |
|  |  |  |  |
|  |  |  |  |
|  |  |  |  |
|  |  |  |  |
|  |  |  |  |
|  |  |  |  |
|  |  |  |  |
|  |  |  |  |
|  |  |  |  |
|  |  |  |  |
|  |  |  |  |
|  |  |  |  |
|  |  |  |  |
|  |  |  |  |
|  |  |  |  |
|  |  |  |  |
|  |  |  |  |
|  |  |  |  |
|  |  |  |  |
|  |  |  |  |
|  |  |  |  |
|  |  |  |  |
|  |  |  |  |
|  |  |  |  |
|  |  |  |  |
|  |  |  |  |
|  |  |  |  |
|  |  |  |  |
|  |  |  |  |
|  |  |  |  |
|  |  |  |  |
|  |  |  |  |
|  |  |  |  |
|  |  |  |  |
|  |  |  |  |
|  |  |  |  |
|  |  |  |  |
|  |  |  |  |
|  |  |  |  |
|  |  |  |  |
|  |  |  |  |
|  |  |  |  |
|  |  |  |  |
|  |  |  |  |
|  |  |  |  |
|  |  |  |  |
|  |  |  |  |
|  |  |  |  |
|  |  |  |  |
|  |  |  |  |
|  |  |  |  |
|  |  |  |  |
|  |  |  |  |
|  |  |  |  |
|  |  |  |  |
|  |  |  |  |
|  |  |  |  |
|  |  |  |  |
|  |  |  |  |
|  |  |  |  |
|  |  |  |  |
|  |  |  |  |
|  |  |  |  |
|  |  |  |  |
|  |  |  |  |
|  |  |  |  |
|  |  |  |  |
|  |  |  |  |
|  |  |  |  |
|  |  |  |  |
|  |  |  |  |
|  |  |  |  |
|  |  |  |  |
|  |  |  |  |
|  |  |  |  |
|  |  |  |  |
|  |  |  |  |
|  |  |  |  |
|  |  |  |  |
|  |  |  |  |
|  |  |  |  |
|  |  |  |  |
|  |  |  |  |
|  |  |  |  |
|  |  |  |  |
